# Editorial: Sensory Abnormalities and Primary Sensory Neurons

**DOI:** 10.3389/fncel.2022.852614

**Published:** 2022-02-04

**Authors:** Takayoshi Masuoka, M. Carmen Acosta, David J. Adams

**Affiliations:** ^1^Department of Pharmacology, School of Medicine, Kanazawa Medical University, Uchinada, Japan; ^2^Instituto de Neurociencias, Universidad Miguel Hernández-CSIC, San Juan de Alicante, Spain; ^3^Illawarra Health and Medical Research Institute (IHMRI), University of Wollongong, Wollongong, NSW, Australia

**Keywords:** dorsal root ganglion, trigeminal ganglion, nociceptor, neuronal excitability, sensory disorder, pain

Primary sensory afferents branching off from the dorsal root ganglia (DRG) and trigeminal ganglia (TG) innervate many tissues including the skin, mucous membranes, and muscles throughout the whole body. These sensory afferents express specific sensors that enable detection of a wide range of stimuli, such as mechanical forces, temperature, and endogenous chemical mediators. The detected information in the periphery triggers nerve impulses that are conducted to the central terminals in the spinal dorsal horn or trigeminal nucleus of the medulla. These signals finally provide warm, cold, touch, pressure, pain, and itch sensations. Therefore, understanding the regulatory mechanisms of excitability and structure of primary sensory neurons and their aberrations is important for bringing the peripheral pathology of sensory abnormalities to light.

Primary sensory nerves are classified into distinct populations (Aδ, Aβ, and C fibers) based on fiber size, myelination and cell body size. Recently, each population has been reported to specifically express a lot of nociceptive and non-nociceptive membrane receptors activated by endogenous and exogenous stimuli. Peterson et al. reported in this Research Topic that physiological levels of lactate mainly released from active muscles can excite group III muscle afferents mediated by hydroxyl carboxylic acid receptor 1 (HCAR1) categorized to a G protein-coupled receptor. They discussed the physiological significance of this lactate sensor in pressor reflex, pain, and muscle fatigue. Unmyelinated C fibers transmitting pain signals have been separated into mechano-sensitive and mechano-insensitive silent classes. The activity of the latter class is thought to be related to the ongoing pain in chronic pain patients. Jonas et al. revealed that the presence of tetrodotoxin-resistant sodium channels that affects axonal calcium mobilization functionally separates mechano-insensitive silent nociceptors from polymodal C nociceptors, although it simply cannot link its conduction and firing properties. Gafurov et al. showed the characteristics of the antidromic spike activity in meningeal afferents. The antidromic firings from TG are weaker than orthodromic firings and are augmented by ATP and capsaicin but not serotonin, whereas the orthodromic firings are strongly potentiated by all compounds. These data would help to clarify the role of antidromic spike flow in migraine in the future. Although most neurophysiological studies in primary sensory neurons have been done in animal models, Hulme et al. generated human sensory neurons differentiated from human pluripotent stem cells (hPSC) by neurogenin-2. The authors characterized the differentiated cells with expressing molecules and membrane excitability and demonstrated that the system is useful for the studies of sensory nerve development, peripheral neuropathies and novel analgesics tests.

Neuropathic pain is one of the most important topics in morbid pain. Recent research has shown the abnormal excitabilities of nociceptor and plasma membrane in addition to morphological abnormalities of primary sensory afferent in nerve injury-induced by chronic constriction and spinal nerve transection or ligation. Acid-sensing ion channels (ASIC) which consist of six transcripts of subunits (ASIC1a, 1b, 2a, 2b, 3, and 4) detects moderate extracellular acidic conditions after injury and inflammation. In this Research Topic, Papalampropoulou-Tsiridou et al. showed the expression patterns of 5 ASIC subunits except for subunit 4 in DRG neurons and clarified the significant alteration of subunits 1a, 1b, and 3 after peripheral nerve injury that induces mechanical hyperalgesia. Membrane excitabilities that are mainly regulated by sodium/potassium balance are also an important factor of neuropathic pain pathology in primary sensory neurons. Smith comprehensively reviewed the dysfunctions of various potassium channels, such as delayed rectifiers, A-type channels, KCNQ or M-channels, Na^+^-activated K^+^ channels, and two-pore domain leak channels, expressed in primary sensory neurons under neuropathic pain. Nerve injury affects not only injured nerves but also neighboring uninjured sensory afferents. Tran and Crawford summarized the variation of uninjured sensory afferents under neuropathic pain from the point of molecular expressions and neurophysiological properties in each fiber type.

This Research Topic reveals new concepts concerning the changes in primary sensory neurons under diseases accompanied by sensory disorder. The ocular surface composed of the cornea and conjunctiva is one of the most densely innervated areas in the body. Guerrero-Moreno et al. reviewed the recent knowledge of corneal nerve morphology and function under sensory abnormalities in corneal nerve injury and dry eye disease, and referred to the neuroimmune interaction in the cornea, TG, and central structure. Masuoka et al. reported that dry eye sensitized transient receptor potential vanilloid 1 (TRPV1)-mediated responses in corneal cold-sensitive nerves and polymodal nerves through the activation of trigeminal satellite cells in the TG, which could contribute to ocular uncomfortableness and pain. Charcot-Marie-Tooth disease type 2D (CMT2D) is a hereditary disease characterized by distal dysfunction of motor and sensory nerves. With a CMT2D model mouse, Sleigh et al. showed specific neurodegeneration of sensory nerves in hindlimbs and no impairment of axonal transport in sensory afferents. Further, the sensory development deficit in CMT2D model mice might link to the elevation of glycyl-tRNA synthetase enzyme in a particular subset of DRG neurons. Endometriosis often causes abdominal pain and chronic pelvic pain especially during menstrual periods, as well as fertility problems. Maddern et al. reviewed endometriosis-induced chronic pain and discussed the mechanisms based on recent clinical and experimental evidence. Muscle spindle afferent (MSA) sensitivity has long been hypothesized to be related to clinical development and/or persistence of muscular pain. To verify the existence of this nociceptive-fusimotor relationship, Lima et al. discussed the results of MSA response to experimentally-induced pain or noxious stimuli in clinical and pre-clinical studies in a systematic review. Regarding pain therapy, prescription opioids are frequently used for alleviating severe pain like cancer and postoperative pain. However, opioids can cause unexpected effects, e.g., opioid tolerance and opioid-induced hyperalgesia. Recently, heteromer of μ and δ opioid receptors and its regulatory molecule receptor transporter protein 4 (RTP4) have been identified with opioid tolerance in the central nervous system. Fujita claimed the possible contribution of these molecules on pain modulation and as a promising target of anti-nociception at primary sensory neurons.

In summary, this Research Topic collects original research and review articles concerning the functions and abnormalities of primary sensory neurons from multiple aspects. These articles show that primary sensory neurons are heterogeneous and with complex phenotypes, which enable them to regulate the various physiological functions and sensations evoked depending on the extracellular conditions. In addition, a variety of alterations and the phenotype switch in primary sensory neurons might be involved in the sensory abnormalities in a wide range of diseases. The increase in this knowledge may help to understand the sensations and sensory abnormalities under pathological conditions, and the development of novel analgesics in the future. We believe that this compilation provides novel knowledge in this research field and helps to spur research that could shed light on mechanisms of sensory abnormalities related to many diseases.

## Author Contributions

TM wrote the editorial. MA and DA critically revised the work and approved its version to be submitted. All authors contributed to the article and approved the submitted version.

## Funding

TM was supported by the grants of JSPS KAKENHI (No. 20K09814), and Smoking Research Foundation of Japan. MA was supported by funding from MICIN/AEI/1013039/5011100011033 (PID2020-115934RB-I00). DA was supported by funding from National Health and Medical Research Council of Australia Program Grant (No. APP1072113).

## Conflict of Interest

The authors declare that the research was conducted in the absence of any commercial or financial relationships that could be construed as a potential conflict of interest.

## Publisher's Note

All claims expressed in this article are solely those of the authors and do not necessarily represent those of their affiliated organizations, or those of the publisher, the editors and the reviewers. Any product that may be evaluated in this article, or claim that may be made by its manufacturer, is not guaranteed or endorsed by the publisher.

